# Potential anti neuro‐inflammatory effect of BioCen‐128 in animal models of dementia

**DOI:** 10.1002/ame2.12267

**Published:** 2022-08-15

**Authors:** Nashelly Esquivel, Yenela García, Roberto Menéndez, Teidy E. García, Ana R. Morales, Claudio Rodríguez

**Affiliations:** ^1^ National Center for Bioproducts (BIOCEN) Bejucal Cuba; ^2^ Center for Neurosciences (CNEURO) Playa Cuba; ^3^ Department of Pharmacology Institute of Marine Sciences (ICIMAR) Havana Cuba

**Keywords:** BioCen‐128, cotton pellet, mice, neuroinflammation

## Abstract

**Background:**

BioCen‐128 is a new active pharmaceutical ingredient composed of a specific bovine thymic fraction of a polypeptide nature. Positive results of similar thymus extracts have been shown to be effective in delaying the processes associated with aging, immunosenescence and Alzheimer's disease (AD), where the inflammation plays an important role. Because of the anti‐inflammatory potential of BioCen‐128, the aim of this study was to evaluate the granuloma model induced by a cotton wool implantation and the model induced by intracerebroventricular (ICV) administration of streptozotocin (STZ).

**Method:**

The experiment was carried out using male OF‐1_cenp_ mice weighing 20 ± 2 g.

**Results:**

Mice administered BioCen‐128 in via the IP route at 5, 10 and 20 mg/kg of corporal weigh showed a decrease in the wet and dry weights of the granuloma, providing evidence of a systemic anti‐inflammatory effect. In the ICV model of STZ, the administration of BioCen‐128 improved cognitive function.

**Conclusion:**

These responses suggested an anti‐neuroinflammatory effect explainable by the action of thymosin β4 and thymosin alfa proteins. The results suggested that BioCen‐128 could be used in the prevention and treatment of some diseases, for example AD, where neuroinflammation is one of the biological events that take place.

## Graphical abstract

BioCen‐128 is a new biological formulation composed of specific bovine thymic fraction of a polypeptide nature. Positive results of similar thymus extracts have shown to be effective in delaying the processes associated with aging, immunosenescence and Alzheimer disease (AD), where the inflammation play an important role. Because of the anti‐inflammatory potential of BioCen‐128, the aim of this study was its evaluation in the granuloma model induced by the implantation of cotton wool and in the model induced by icv administration of STZ. In the i.c.v model of STZ, the administration of BioCen‐128 improvement the cognitive function. These results suggested that BioCen‐128 could be used in the prevention and treatment of some diseases where neuroinflammation is one of the biological events that take place, for example in AD.

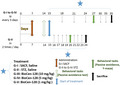

## 1 INTRODUCTION

Inflammation is a transient biological response of vascularized tissues to a noxious stimulus such as injury, or exogenous or endogenous antigens, designed to eliminate the stimulus and repair damaged tissue.^1^ Although inflammation is a positive defense mechanism, a prolonged and uncontrolled reaction can result in different disorders and pathologies.^2^, ^3^ Inflammation is a very complex physiological response with two phases: one acute and the other chronic.^4^ The first is characterized by the presence of vascular changes, the infiltration of leukocytes and the release of inflammatory mediators responsible for the transition of inflammation from an acute to a chronic phase.^5^, ^6^ In chronic inflammation, cell damage occurs with the simultaneous destruction and healing of damaged tissue,^7^ during which cyclooxygenase (COX) is activated to produce prostaglandins (PGs), responsible for pain, edema and fever.^8^ Acute inflammation is a key element in the systemic inflammatory response syndrome, considered a clinical entity always secondary to an underlying pathology. Long‐term activation in the innate immune system, a product of a set of individual alterations of risk factors, constitutes a unified mechanism capable of triggering an inflammatory cascade that results in alterations in the cytoskeleton with neurodegenerative consequences.^9^ One of these is neuroinflammation, a brain response designed to inactivate or eliminate potential damage that is mediated by the glia and the lymphocytes, monocytes and macrophages of the hematopoietic system. In the pathology of neurodegenerative diseases such as Alzheimer's disease (AD), the joint action of chronic neuroinflammation and pathological aging plays a fundamental role.^10^ Regulation of innate immunity and phagocytosis have been identified as risk factors for AD. Neuropathological studies have shown that the neuroinflammation process in the disease triggers a self‐perpetuating cycle that activates microglia and favors the release of inflammatory cytokines in the cerebral cortex.^11^, ^12^, ^13^

Considering the adverse reactions that appear after prolonged use of synthetic anti‐inflammatory drugs it is important to search for effective natural agents with low toxicity and better tolerability.^14^ Furthermore, there is no reference to the fact that there are currently approved drugs whose therapeutic target is neuroinflammation associated with AD. However, 33 products are reported in different phase of clinical evaluation with this therapeutic target, of which three are proteins in nature.^15^

Positive results have been reported for thymic preparations or individual thymic proteins whose effect is to reduce neuroinflammation and/or act as immunomodulators. It has been reported that although these preparations or proteins can act by improving acquired immunity, the vast majority act on cellular immunity by modulating the production, maturation and activation of T lymphocytes (LT) and macrophages^16^, ^17^, ^18^ and helping to restore the immunological response to different pathophysiological states.

One of the inflammation models that has been used to evaluate the anti‐inflammatory effect of certain compounds in chronic inflammation is that of a granuloma induced by the subcutaneous implantation of a sterile cotton pellet. This is one of the most widely used models for the detection of compounds that act on chronic inflammation and specifically those in the earlier stages of its development in which the migration and activation of inflammatory cells have a primary role.^19^ On the other hand, a model that involves the intracerebroventricular (ICV) injection of streptozotocin (STZ) is used to alter insulin signaling in the brain and favor the development of neuroinflammation and deterioration in cognitive function. In addition, this model allows the development of neurofirilla tangles by hyperphosphorylation of tau protein and the formation of amyloid plaques.^10^

The aim of this study was to evaluate the potential anti‐inflammatory effect of BioCen‐128 in two models: (i) the granuloma model induced by the implantation of cotton wool designed to model systemic inflammation and (ii) the model induced by ICV administration of STZ where the neuroinflammation is present.

## 2 MATERIALS AND METHODS

BioCen 128 is a sterile active pharmaceutical ingredient (API) prepared at a concentration of 1.7 mg/ml of total proteins, dissolved in 0.9% NaCl solution. The API was obtained on an industrial scale in the Active Ingredient Production Plant under Good Manufacturing Practice at the Centro Nacional de Biopreparados with a density of 1.002 g/ml. BioCen 128 is a purified extract of bovine thymus containing thymosin α1 (Tα1) and thymosin β4 (Tb4) as major components. The extract also contains ubiquitin and other minor thymus derived proteins involved in the modulation of immune system, in the synapsis and neuronal plasticity, and the synthesis and conformation of proteins, among them ubiquitin fold modifiers, cytochrome C, splicing and elongation factors, histones and cathelicidins.^20^ BioCen‐128 should be stored at 2–8°C.

### 2.1 Thymosin quantification

An ELISA kit (Immunodiagnostik AG, Germany) was used for the quantitative in vitro determination of Tα1 in BioCen 128. Rabbit polyclonal antibodies directed against synthetic Tα1 were used in this enzyme immunoassay for the determination of Tα1. The principle of the test is based on a competition between the antigen in the sample or standards and the antigen coated in the wells of the microplate. A peroxidase‐conjugated antibody is used for detection and quantification, and tetramethylbenzidine (TMB) is used as a peroxidase substrate. The enzymatic reaction was terminated with an acid stop solution. In the case of the quantification of Tb4, another set of reagents from the same supplier was used, with a principle similar to the previous one. In this determination, a curve of different concentrations of Tb4 (0 to 10 μg/ml) was used to quantify the thymosin in the sample from the ABS reading at 450 nm in a microplate reader. In addition, two samples of known concentration were used as positive controls, whose expected concentration range is found in the manufacturer's certificate.

### 2.2 Animals and housing conditions

OF‐1_cenp_ male mice were purchased from the National Center for Laboratory Animal Breeding (CENPALAB), with an average weight at the beginning of the experiment of 20 ± 2 g. The mice were kept in a room with a controlled temperature of 20 ± 3°C and light–dark cycles of 12 h each. They were provided with food and water on demand and were adapted for 7 days before the experiments began under the established conditions. Subsequently, the ears were pierced, allowing their individual identification according to established procedures. The mice were housed in polypropylene cages with 5 animals per box.

### 2.3 Experimental protocol

#### 2.3.1 Cotton pellet‐induced granuloma

Cotton pellet‐induced granuloma in mice was achieved according to the method described by Bertollo et al.^21^ Granulomatous lesions were induced by surgically implanting sterile cotton pellets (10 ± 0.5 mg) subcutaneously in the dorsal region of the mice grouped into 5 treatment groups of 10 animals. Group I had vehicle administered with 0.9% sterile saline solution intra‐peritoneally (IP); group II was a positive control administered with dexamethasone (3 mg/kg body weight), orally; and groups III, IV and V were treated with BioCen‐128 IP (20, 10 and 5 mg/kg body weight). All treatments were given once daily for 7 consecutive days. On day 8, the mice were anesthetized, and the pellets covered by granulomatous tissue were dissected out. The weight of the wet and dry granulomas were calculated (mg × 100) and used for statistical analysis. The inhibition percentages (% Inh) were calculated for the wet and dry weights using the following expression:

$$\%\mathrm{Inh}=\left(X-Y\right)/X\times 100,$$

where *X* is mean value of the weight (wet or dry) of the cotton pellets of the control group and *Y* is mean value of the weight (wet or dry) of the cotton pellets of the treated group. The mean weights for different groups were determined, and compared to the control group.

#### 2.3.2 Intracerebroventricular (ICV) infusion of streptozotocin (STZ)

After 7 adaptation days, mice were anesthetized with a mixture of ketamine (Sigma‐Aldrich, Germany; 100 mg/kg body weight) and xylazine (Sigma‐Aldrich, Germany; 5 mg/kg body weight) IP and placed on a stereotaxic frame. The skull was then exposed in the vertical and horizontal planes. Using a scalpel, an incision was made on the skin from the anterior corner of the eyes to the beginning of the neck muscles. After laterally separating the periosteum, the shell was exposed and the Bregma point was located as a reference point for the location of the stereotaxic coordinates.^22^ The STZ was dissolved in a fresh solution of artificial cerebrospinal fluid (SACF) and injected bilaterally (1.5 mg/kg body weight) ICV, in a maximum volume of 10 μl, according to the method of Haley and McCormick.^23^ The sham group underwent the same surgical procedures, but with the same volume of SACF injected instead of STZ.

The mice were grouped in 5 treatment groups of 10 animals. Group I had vehicle administered with SACF ICV, and saline as treatment; group II was a positive control administered with STZ ICV and saline as treatment; groups III, IV and V were administered with STZ ICV, and treated with BioCen‐128 (10 and 5 mg/kg body weight) IP and (1 mg/kg body weight) intra‐nasally (IN).

**FIGURE 1:**
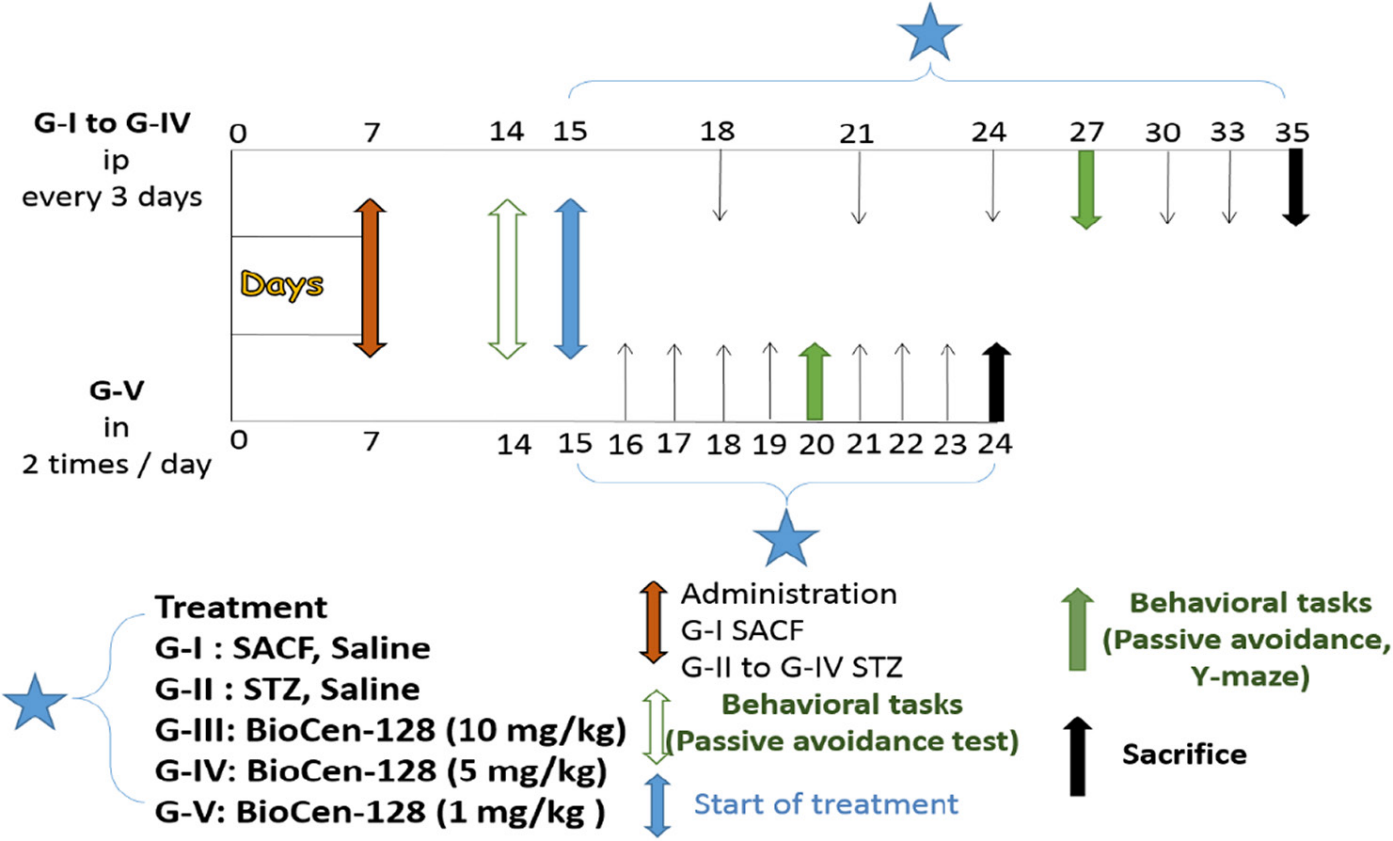
Schematic representation of the experimental design used

The doses of BioCen‐128 used were 10 and 5 mg/kg body weight, IP every 3 days. The group to which BioCen‐128 was administered IN received a dose of 1 mg/kg body weight daily at a rate of 5 μl in each nostril/2 times/day. After 5 administrations IP and 12 IN with BioCen‐128, analysis of the treatment effect on behavioral tests was started, maintaining the dosage regimen of the different groups (Figure 1).

### 2.4 Behavioral tasks testing

The behavioral tasks were only applied in the STZ model. Animals were adapted for 24 h before the start of the behavioral tests. Throughout the execution of the tests, the equipment was disinfected with a 70% ethanol solution and the researcher who performed the tests was blind to the experimental protocol.

#### 2.4.1 Passive avoidance test

In this study, the mice were tested for memory retention deficits using a passive avoidance apparatus following the methodology described by García and Esquivel^24^ at two different time points. At 14 days after the administration of STZ, the establishment of cognitive deficit was confirmed using the passive avoidance test. Those animals that did not present cognitive deficit were eliminated from this study. In addition, at a second time point after treatment with BioCen‐128, the effect of treatment was evaluated using the same behavioral test.

A box with two compartments separated by a guillotine door was used for this test. The test began by placing the mouse in an illuminated compartment for the acquisition phase, allowing an adaptation period of 25 s. After this time, the door was released so that the mouse could pass into a dark compartment with a stainless steel mesh floor. The initial latency of the animals entering the dark chamber was recorded and those that exceeded 60 s were excluded. Once the mouse was inside the dark chamber, the door was closed and an electrical discharge of 0.3 mA for 5 s was delivered before the mouse was returned to its cage. Retention latency, up to a maximum of 300 s, was measured at 24 h in the same way as in the acquisition test but without the electric shock. The latency time was recorded.

#### 2.4.2 Y‐maze task (one arm blocked)

The Y‐maze task allows evaluation of the spatial memory of short‐term work and is based on the natural instinct of rodents to explore unknown environments. This trial was performed only to assess the effect of BioCen‐128 treatment after cognitive impairment had been established. Each arm was identified by a different letter (A, B, C), following the methodology described by García and Esquivel.^24^

The C arm of the Y‐maze was blocked and the mouse was placed in the center of the maze to freely explore A and B arms for 7 min. Mice that did not show exploratory activities during the first 2 to 3 min of the training phase of the task were rejected. After 1 h, the retention phase started. The animal was returned to the maze to explore all three arms freely for another 7 min. The behavior of the animal during the second part of the test (three arms free) was recorded with a non‐professional digital camera.

The percentage of time that every animal visited each arm was calculated from the total time of the task by the following expression:

$$\text{Percentage of time spent}\;\mathrm{on}\;\text{arms}\;A,B\;\text{or}\;C\;\left(\%\right)=\frac{\text{Time spent visiting}\;\mathrm{arm}\;\left(s\right)}{\text{Total time to visit the three arms}\;\left(s\right)}\times 100$$

### 2.5 Statistical analysis

Results are expressed as means ± SEM (*n* = 10). GraphPad software version 5.0 was used (USA, 2007). All variables were analyzed using the Kolmogorov–Smirnov test to check the normality. Variance homogeneity was analyzed using Fisher's test. Statistical analysis of the data was obtained by applying a Student's *t* test of independent samples or by an analysis of variance (ANOVA), followed by Tukey's test, when more than two samples were analyzed. *p* < 0.05 was considered statistically significant.

## 3 RESULTS

### 3.1 Quantification of thymosins

The ELISA results obtained for both the major components of the purified thymic extract (Tb4 and Tα1 proteins) were analyzed for two batches of BioCen‐128. The concentration of Tb4 was 21.71 ± 1.03 ng/ml and that of Tα1 was 218.3 ± 17.4 ng/ml.

### 3.2 Cotton pellet‐induced granuloma

**FIGURE 2:**
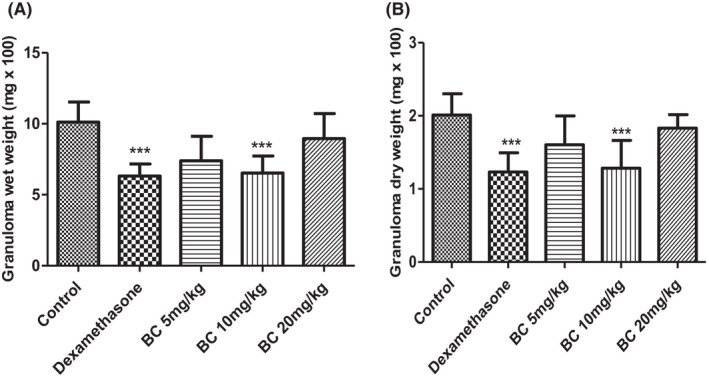
Effects of the administration of BioCen‐128 (BC) at doses of 5, 10 and 20 mg/kg body weight in the cotton pellet‐induced granuloma model based on implantation of a cotton pellet subcutaneously in the dorsal region of the mice. (A) Wet weight. (B) Dry weight. Data (*n* = 10) shown are the means ± SD. *p < 0.05 compared to control group

The IP administration of BioCen‐128 at doses of 5 and 10 mg/kg showed a gradual decrease in the wet and dry weights of the granuloma compared to the control group (Figure 2). The best response was obtained with a dose of 10 mg/kg body weight (wet weight: 38.87%; dry weight 37.15%, *p* < 0.05), and was similar to that obtained with the positive control (dexamethasone). However, the administration of dexamethasone, the reference substance, also reduced the weight of the granuloma (*p* < 0.05) by a magnitude of 40.83% for wet weight and 39.40% for dry weight. While at a dose of 20 mg/kg, a behavior similar to the negative control group was obtained, without a significant decrease in the wet and dry weights of the granuloma.

The establishment of inflammation and the formation of the granuloma is a gradual process that lasts about 7 days, during which the proliferation, migration and infiltration of inflammatory cells, macrophages and neutrophils occurs initially towards the area where the inducing agent has been implanted.^19^ As well as changes to fibroblasts, a process of inflammatory angiogenesis is also induced^25^ along with an increase in cytosines, chemokines, eicosanoids and oxidative stress.^26^

### 3.3 Intracerebroventricular (ICV) infusion of streptozotocin (STZ)

**FIGURE 3:**
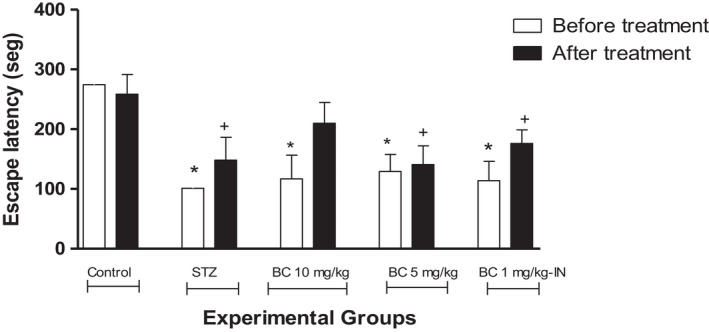
Effect of BioCen‐128 (BC) in animals showing cognitive deficit in the passive avoidance test induced by STZ at 1.5 mg/kg body weight. *p < 0.05 compared to control group before treatment, indicating that the animals start from a similar cognitive deficit between the groups; ^+^ *p* < 0.05 compared to control group after treatment

The establishment of the model of cognitive deficit by ICV administration of STZ allowed the study of BioCen‐128 in a model of AD where neuroinflammation is one of the most exacerbated events.^27^ In this experiment, doses of 5 and 10 mg/kg administered by the IP route were evaluated, being the doses that showed the best results in the previous experiment. The IN route was also evaluated at a dose of 1 mg/kg. Cognitive function was evaluated by the passive avoidance test (Figure 3) in the animals before and after treatment. The animals that received STZ showed cognitive impairment, having a significantly lower escape latency than the control group. However, after treatment with BioCen‐128, only the group that received the 10 mg/kg body weight dose showed no significant differences in relation to the controls for this variable. The rest of the treated groups had significantly lower escape latencies than the control group.

**FIGURE 4:**
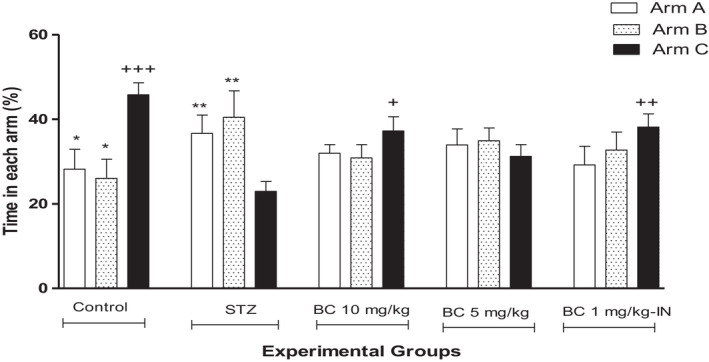
Effect of BioCen‐128 (BC) in animals showing cognitive deficit in the Y‐maze test induced by STZ at 1.5 mg/kg body weight; each value represents the percentage time spent in each arm during the 5 min of the retention test. *p < 0.05, **p < 0.01 compared to arm C, the novel arm, within each group; ^+^ *p* < 0.05, ^++^ *p* < 0.01, ^+++^ *p* < 0.005 compared to arm C for the STZ group)

The closed arm Y‐maze test (Figure 4) was the other behavioral task used. Both the group that was administered with BioCen‐128 IP at a dose of 10 mg/kg body weight and the group that received this formulation IN showed an improvement in cognitive function. In these cases, the time spent in the blocked arm (C) was significantly greater than in the other two (A and B).

## 4 DISCUSSION

The anti‐inflammatory mechanism proposed for BioCen‐128 is based on the action of its major components, Tb4 and Tα1. The presence of Tb4 has a stimulating effect on lymphocyte (LT) differentiation by inducing the expression of the terminal deoxynucleotidyl‐transferase. It also inhibits macrophage migration and has effects on the hypothalamus and the pituitary gland.^28^ The cross‐linking of Tb4 or other beta thymosins is probably a general mechanism for binding to extracellular structures such as fibrin and collagen and its action as a chemotactic or regulator of the inflammatory response at both the systemic and cerebral levels.^28^, ^29^ The regulation of infiltration into the central nervous system (CNS) of CD4+T cells, which occurs through adhesion molecules and ligands present in the blood–brain barrier, plays a fundamental role in the transition between the neurodegenerative and neurosuppressive phenotypes of the microglia.^30^ Thus, the predominance of one or other pathway will depend on the numerical and functional predominance of the T cells present, whether they are effector or regulatory. In addition, Tb4 contributes to the preservation of spatial memory under physiological conditions.^31^ With the supposed decrease in neuroinflammation mediated by LTs, restitution of the synapses between neurons may have occurred, thus improving the cognitive function of the animals treated.

Tα1, on the other hand, is an active 28 amino acid polypeptide, originally isolated from thymic tissue as one of the compounds responsible for reconstitution of immune function in thymectomized animal models.^32^ It has been shown to act through Toll‐like receptors binding to intracellular cell signaling pathways, causing stimulation of the immune system, including T helper, cytotoxic T cells, and natural killer cells.^33^ Studies have shown that thymectomy impairs learning and memory in mice, while Tα1 modulates excitatory synaptic transmission in cultured hippocampal neurons.^34^ Both, thymosins produced in the thymus and those which can be administered peripherally activate the immune system to positively regulate neurogenesis, neuroprotection and promote learning, memory, and hippocampal long‐term potentiation.^35^ According to a recent report, based on Tβ4 potential anti‐inflammatory molecules and neuroprotective and myelin regeneration molecules and their mechanism of action, Tα1 and Tb4 could be used alone to treat multiple sclerosis (MS) or with other approved applications of drug combination therapy.^29^

In the experiment using cotton pellet‐induced granuloma, the decrease in the wet and dry weights (Figure 2) in the group treated with BioCen‐128 at a dose of 10 mg/kg in relation to the control is a positive result. In this model, the increase in the dry weight of the cotton pellet is considered as a measure of granuloma formation.^36^ The fact that BioCen‐128 significantly reduces granuloma formation suggests that its anti‐inflammatory activity resides primarily in the proliferative phase of the inflammatory process. In this model, the formation of granuloma begins with the presence of the antigen (cotton pellet), causing stimulation of the immune system, which under persistent antigenic stimulation contributes to the proliferation and formation of granulomatous tissue around the cotton pellet‐induced granuloma.^37^ Therefore, the granuloma is a focus of chronic inflammation consisting of the microscopic aggregation of macrophages that transform into epithelial‐like cells surrounded by a collar of mononuclear leukocytes, mainly lymphocytes and occasionally plasma cells.^38^

The anti‐inflammatory effect of BioCen‐128 could therefore be explained by inhibition of the release of arachidonic acid by macrophages that could result in a lower accumulation of fluid and plasma protein material, less infiltration of neutrophils in the cotton pellet and therefore, a lower weight of the granuloma in the treated animals.^19^ Alternatively, BioCen‐128 could cause a decrease in the influx of T lymphocytes to the granuloma site and therefore a decrease in cytokines released by these cells (TNF‐α, IL‐2 and IFN‐α); this in turn affects the transformation and proliferation of macrophages in epithelial cells, leading to the decrease in the weight of the granulomatous tissue.^38^ These actions are due to the thymic proteins that the formulation contains, as mentioned above.

The dose‐dependent effect of BioCen‐128 does not follow a typical dose–response relationship in sigmoid form, but rather has a U‐shaped behavior. This type of non‐monotonic or biphasic pharmacological responses is found within the so‐called hormesis‐type pharmacological responses, which can be produced by the appearance of adaptation mechanisms within the pharmacological response studied^39^ and have been described for compounds of natural origin and for mixtures of several active principles in which several targets and mechanisms of action can converge.^40^

Considering the second experiment, several studies have shown that the ICV administration of STZ in rodents is one of the animal models most compatible with the inflammatory hypothesis of AD because it manages to reproduce the causes, symptoms and lesions in a chronological order very similar to those observed in this pathology. In this experiment a neurobehavioral study with two behavioral tasks was carried out. In addition, following the results obtained with the granuloma model it decided to try an IN route at a dose of 1 mg/kg because of its advantageous characteristics.

At the two doses of BioCen‐128 tested by the IP route in the STZ model, an increase in the latency period was observed in the passive avoidance test (Figure 3). An increase in the residence time in the closed arm of the Y‐maze was also observed (Figure 4). For this reason, the comparison with the cognitive deficits group suggests that this formulation has anti‐neuroinflammatory potentialities. The effects on cognitive improvement obtained in this study were related to the dose tested, being more significant at the maximum dose tested (10 mg/kg body weight). In the case of the IN administration trial, a relative improvement in behavioral parameters was also observed. This experiment was carried out at a lower dose than those used for the IP route because the nasal route presents advantages in relation to the possible greater bioavailability of the components of BioCen‐128 in the CNS. The nasal mucous is one of the most permeable areas in the body and therefore the intranasal route of administration could provide a higher probability for proteins and peptides to reach the CNS,^41^ suggesting that this route is promising for continuing studies. Reinforcing this idea is the fact that thymus proteins, especially Tα1 and Tb4, have been shown to cross the blood–brain barrier.^28^, ^35^ With the supposed decrease in neuroinflammation mediated by LTs, restitution of the synapses between neurons may have occurred, thus improving the cognitive function of the animals treated with the BioCen‐128 formulation in the STZ model.

To confirm this potential effect, further experiments should be performed to assess relevant inflammatory factors (example, TNF‐α, IL‐2 and IFN‐α) and provide histological evidence.

## 5 CONCLUSIONS

Based on the composition of BioCen‐128, two hypotheses are proposed to explain its anti‐inflammatory mechanism. The first hypothesis is based on the action of Tβ4 as a modulator of the immune system. The second hypothesis is based on modulation of neuroinflammation through the action of Tɑ1, another modulator protein of the immune system active in reconstitution of immune function.

The anti‐inflammatory properties of BioCen‐128 were demonstrated using a cotton pellet‐induced granuloma, with the drug administered via the IP route. In this experiment the effects observed showed that the optimal dose of BioCen‐128 for carrying out cognitive deficit tests in this model was 10 mg/kg body weight.

Administration of BioCen‐128 in mice by the IP and IN routes in the Alzheimer's model induced by the administration of STZ improved cognitive function. BioCen‐128 could potentially be used in the prevention and treatment of AD, where neuroinflammation is one of the biological events that are present.

## AUTHOR CONTRIBUTIONS

Nashelly Esquivel Crespo (30%) and Yenela García Hernández (25%) designed and performed experiment; organized and analyzed data wrote and correct the manuscript. Roberto Menéndez Soto (10%), Teidy García (10%) and Ana Ruth Morales (10%) conducted experiments. Claudio Rodríguez Martínez (15%) managed general research, revised, corrected and approved the manuscript as project leader. All authors read and approved this final manuscript.

## CONFLICT OF INTEREST

The authors declare that they have no competing interests to publish these results.

## ETHICS STATEMENT

All the animal care procedures were approved by the Institutional Animal Care and Use Committee at National Center for Bioproducts (BioCen, Mayabeque, Cuba) and were performed according to guidelines compliant with current international laws and policies (NIH *Guide for the Care and Use of Laboratory Animals*, NIH Publication No. 85‐23, 1985).

## FUNDING STATEMENT

None funding was received.
